# *I*_f_ Channel as an Emerging Therapeutic Target for Cardiovascular Diseases: A Review of Current Evidence and Controversies

**DOI:** 10.3389/fphar.2017.00874

**Published:** 2017-11-24

**Authors:** Hayelom G. Mengesha, Tadesse B. Tafesse, Mohammed H. Bule

**Affiliations:** ^1^Pharmacology and Toxicology Research Unit, School of Pharmacy, Mekelle University, Mekelle, Ethiopia; ^2^College of Medicine and Health Science, Adigrat University, Adigrat, Ethiopia; ^3^School of Pharmacy, College of Health and Medical Sciences, Haramaya University, Harar, Ethiopia; ^4^Department of Pharmacy, College of Medicine and Health Sciences, Ambo University, Ambo, Ethiopia

**Keywords:** cardiovascular diseases, heart rate, *I*_f_ channel, ivabradine, therapeutic target

## Abstract

In 2015, non-communicable diseases accounted for 39.5 million (70%) of the total 56.4 million deaths that occurred globally, of which 17.7 million (45%) were due to cardiovascular diseases. An elevated heart rate is considered to be one of the independent predictors and markers of future cardiovascular diseases. A variety of experimental and epidemiological studies have found that atherosclerosis, heart failure, coronary artery disease, stroke, and arrhythmia are linked to elevated heart rate. Although there are established drugs to reduce the heart rate, these drugs have undesirable side effects. Hence, the development of new drugs that selectively inhibit the heart rate is considered necessary. In the search for such drugs, almost four decades ago the *I*_f_ channel, also known as the “funny channel,” emerged as a novel site for the selective inhibition of heart rate. These *I*_f_ channels, with a mixed sodium and potassium inward current, have been identified in the sinoatrial node of the heart, which mediates the slow diastolic depolarization of the pacemaker of the spontaneous rhythmic cells. The hyperpolarization-activated cyclic nucleotide-gated (HCN) subfamily is primarily articulated in the heart and neurons that are encoded by a family of four genes (HCN1-4) and they identify the funny channel. Of these, HCN-4 is the principal protein in the sinoatrial node. Currently, funny channel inhibition is being targeted for the treatment and prevention of cardiovascular diseases such as atherosclerosis and stroke. A selective *I*_f_ channel inhibitor named ivabradine was discovered for clinical use in treating heart failure and coronary artery disease. However, inconsistencies regarding the clinical effects of ivabradine have been reported in the literature, suggesting the need for a rigorous analysis of the available evidence. The objective of this review is therefore to assess the current advances in targeting the *I*_f_ channel associated with ivabradine and related challenges.

## Introduction

A total of 56.4 million deaths occurred globally in 2015, of which non-communicable diseases (NCDs) were responsible for 39.5 million cases (70%) and 30.7 million cases occurred in low- and middle-income countries, for which approximately 48% of the deaths took place before the age of 70. Of these NCD death cases, cardiovascular diseases (CVDs) were responsible for 17.7 million (45%) of the deaths (WHO, 2014, 2016). Based on data from the World Health Organization (WHO), the total annual number of deaths from NCDs is projected to increase to 52 million by 2030 (WHO, 2014). The most well-known risk factors for CVDs include elevated blood pressure, smoking, high cholesterol and blood glucose level, poor diet, obesity, and risky use of alcohol (WHO, 2011, 2014). However, recent studies have shown that the resting heart rate is one of the major indicators of CVD morbidity and mortality in addition to the previously known factors (Reil et al., 2011). The occurrence of atherosclerosis, coronary artery disease (CAD), heart failure, hypertension, and stroke are linked with an elevated heart rate, independently of the other CVDs (Kannel et al., 1987; Gillman et al., 1993; Diaz et al., 2005; Custodis et al., 2008, 2010; Fox et al., 2008).

The occurrences of coronary atherosclerosis in patients that have undergone coronary artery bypass surgery or recurring myocardial infarction have been independently linked to intrinsically elevated heart rate as a result of increasing the mechanical load and tensile strength on the arterial wall and exposure to low endothelial shear stress (Custodis et al., 2008; Lang et al., 2010).

To date, beta blockers, calcium-channel blockers, and other drugs have been utilized to reduce the heart rate either directly or indirectly (Parker and Parker, 1998; Freemantle et al., 1999; Poole-Wilson et al., 2004; Speranza et al., 2012). These medications have been prescribed for heart failure, angina, and other CVDs. Even though these classes of drugs have irreplaceable use in a myriad of CVDs, they have been claimed to exhibit a lack of selectivity in the reduction of heart rate. They also have additional undesirable side effects and contraindications on the respiratory system, angioedema, metabolism, and other sites.

Taking the limitations of the aforementioned classes of drugs into consideration, researchers have investigated a novel target site called the funny current (*I*_f_) or funny (f) channel, which may be useful for selectively lowering the heart rate. This site in the mammalian sinoatrial node (SAN) has been described as the pacemaker of the heart, and it is activated in phase 4 of the action potential as a result of accelerating diastolic depolarization (Speranza et al., 2012).

**FIGURE 1:**
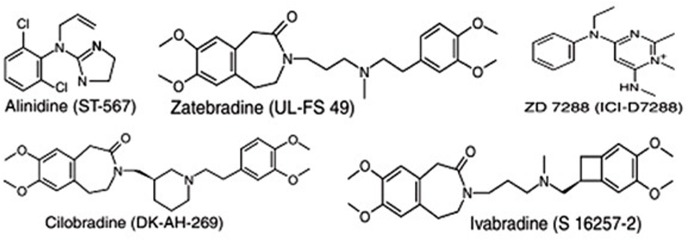
Chemical structures of some “heart-rate-lowering” agents targeting the pacemaker channels of the sinus node.

A number of molecules have been identified and developed as *I*_f_ channel blockers, including alinidine (ST-567), cilobradine (DK-AH-269), zatebradine (UL-FS-49), ZD7288 (ICI-D7288), and ivabradine (S-16257-2) (**Figure 1**). However, ivabradine (a benzocyclobutene-containing compound) is the only molecule that has passed all of the clinical trial phases and that is used currently as an anti-anginal drug (Bucchi et al., 2007).

Overall, this novel site has been continuously investigated for the treatment of a variety of CVDs since its identification almost four decades ago. However, several controversies have arisen in the literature from both experimental animal studies and randomized controlled trials, as well as from a meta-analysis on previously developed drugs that target the *I*_f_ channel, particularly ivabradine (Borer et al., 2003; Fox et al., 2008; Tardif et al., 2009; Cammarano et al., 2016), even though these drugs have been approved by some regulatory agencies and applied in some countries (FDA, 2015). Therefore, the aim of this review is to assess the current evidence and progress on targeting the *I*_f_ channel to determine the future direction and challenges for both preventive and therapeutic purposes.

## *I*_f_ Channel and Its Role

In 1979, mammalian SAN cells, a specialized region of the heart, were found to express the funny channel as an inward current triggered on hyperpolarization in the diastolic range of voltages, thus influencing the contraction rate of the entire heart (Brown et al., 1979; DiFrancesco, 2008).

The name “funny current” arose because of its numerous unusual characteristics, including the mixed Na^+^ and K^+^ current permeability, activation on hyperpolarization, and slow activation and deactivation kinetics (Brown and Difrancesco, 1980; DiFrancesco, 1993; Baruscotti et al., 2005). Studies have shown that the *I*_f_ channel is sensitized at about -100 to -110 mV, and its reversal potential is about -10 to -20 mV, which indicates that it has a mixed permeability to Na^+^ and K^+^. Hence, it is defined as an inward current activated during hyperpolarization at voltages in the range of diastolic depolarization, and it contributes to the generation of rhythmic cardiac activity (Brown and Difrancesco, 1980; Freemantle et al., 1999). Another unusual feature of the *I*_f_ channel is its direct activation by cyclic adenosine monophosphate (cAMP), which activates the opening probability of the *I*_f_ channel, and it is also regulated by voltage-dependent activation (DiFrancesco and Tortora, 1991). Thus, it is evident that its function, and hence the heart rate, is influenced by the concentration of the second messenger cAMP, which will be increased and decreased by adrenergic and cholinergic stimulation, respectively.

**FIGURE 2:**
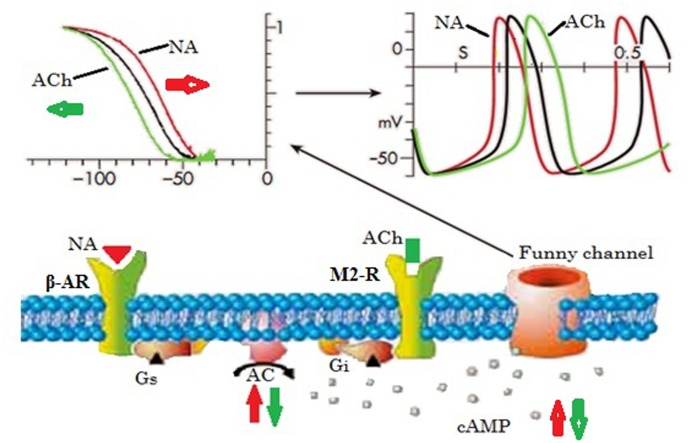
Basic properties of the funny current and its role in autonomic heart rate modulation by up- or down-regulation of cellular cAMP. Elevated cAMP shifts the voltage dependence of the funny channel activation curve to the right, thus increasing current availability during diastole, hence diastolic rate, while the opposite occurs when cAMP is lowered. Ach, acetylcholine; AC, adenylate cyclase; cAMP, cyclic adenosine monophosphate; Gi, inhibitory regulative G-protein; Gs, stimulatory regulative G-protein; β-AR, β-adrenoceptor receptor; NA, noradrenaline; M2-R, type-2 muscarinic receptor (Adapted from DiFrancesco and Borer, 2007 with permission).

As shown by the overview in **Figure 2**, the *I*_f_ activation range (black line in the top left figure) comprises the range of diastolic (pacemaker) potentials (-120 mV to ∼-40 mV), and determines the slope of diastolic depolarization, and hence the rate of the heart, under controlled conditions (black line in the top right figure). Noradrenaline causes stimulation of G proteins by stimulating the β-adrenoreceptors and hence increases the adenylate cyclase activity (red arrow), which in turn increases the intracellular cAMP concentration, and this results in extra activation of *I*_f_ by changing the *I*_f_ activation curve to more positive voltages (red line in top left figure). Thus, more inward current brings about a short diastolic depolarization period, and hence the heart rate speeds up (red line in top right figure) (DiFrancesco and Borer, 2007).

Sinoatrial node cells in the myocardium are distinguished by the incidence of a “slow diastolic” phase that produces an impulsive and recurring action potential (DiFrancesco, 1993). Currently, it is generally understood that the generation of the diastolic depolarization is caused by activation of the *I*_f_ channel at the end of an action potential. Therefore, either stimulation or inhibition of the *I*_f_ channel may trigger or suppress the heart beat by altering the diastolic time of depolarization. Hence, the activation of the *I*_f_ channels decreases the diastolic time, which subsequently produces myocardial and associated problems. Alternatively, the inhibition of the *I*_f_ channels increases the diastolic time because of the slower firing of the next action potential and beat. This, in turn, results in improved perfusion of the myocardium and reduces its oxygen demand, which is beneficial for the management and prevention of CAD, heart failure, and other CVDs.

Studies conducted prior to the identification of the *I*_f_ channel concluded that the enhancement of the K^+^ conductance of the pacemaker cells in the SAN due to the discharge of acetylcholine (Ach) slows the heart rate (Rayner and Weatherall, 1959; Danilo et al., 1978). In addition, an experimental study by Sakmann et al. (1983) suggested that K^+^ channels are responsible for the ACh-dependent K^+^ conductance increase in SAN cells, which possess distinct opening and conductance properties compared to atrial and ventricular cells. However, SAN cells have similar properties to the other ACh-activated cells in terms of resting K^+^ current. This study also found an excess of short channel openings, the reason for which was unclear (Sakmann et al., 1983) but could be the occurrence of *I*_f_ channel activation, although it was not considered in this study. After this finding, a study by DiFrancesco et al. (1989) revealed that at low (resting) or moderate vagal activities, ACh-mediated *I*_f_ current inhibition is responsible for the slowing of heart rate, while the K^+^ current is activated by a marked vagal stimulation (high ACh concentration). The result shows in a pronounced decrement of heart rate (bradycardia) in the SAN cells and atrium as well (DiFrancesco et al., 1989; DiFrancesco and Borer, 2007). These findings provide new insights into the mechanism through which the heart rate is decreased upon activation of muscarinic receptors by ACh (green lines, as shown in **Figure 2**).

## HCN Channel: The Molecular Components of the *I*_f_ Channel

The mammalian hyperpolarization-activated cyclic nucleotide-gated (HCN) channel family consists of four isoforms, namely HCN1–4, which belong to the superfamily of voltage-gated potassium channels and represent the molecular α subunits of the native “funny” channels present in the heart and brain (Baruscotti and Difrancesco, 2004; Baruscotti et al., 2010a,b; Scicchitano et al., 2012). *In vivo* examinations have revealed that functional HCN channels can be produced in homomeric as well as heteromeric tetramers with the exception of HCN2–3 heteromers (Baruscotti et al., 2010a). The HCN4 isoform is the dominant HCN transcript in human SAN cells, followed by HCN1 and HCN2, although HCN2 is the foremost transcript in ventricles (Chandler et al., 2009; Scicchitano et al., 2012) and its locus has been recognized as a modulator of heart rate in a genome-wide association study (den Hoed et al., 2013).

The physiological role of the HCN family of channels in the central nervous system and heart is enormous (DiFrancesco and Tortora, 1991; Baruscotti et al., 2010a), and they are responsible for the *I*_f_ current in the SAN. Six-transmembrane α-helical segments (S1–S6) make up each HCN isoform, with the positively charged S4 domain acting as a voltage sensor in the pore region formed between domains S5 and S6, which acts as a conduction pathway and selectively filters binding. The presence of a glycine–tyrosine–glycine (GYG) sequence, which is typical for the pore-loop region of K^+^-permeable voltage-gated ion channel subunits, has been reported; a linking site for cAMP in the C-terminal region of the peptide is found that binds to the channels, through a direct action on the channel itself but not by phosphorylation, in the cyclic nucleotide-binding domain (CNBD) located within the C-terminus (Barbuti et al., 2007; Baruscotti et al., 2010a; Scicchitano et al., 2012). The binding of cAMP to the HCN channels increases the probability of the channel to be open during hyperpolarization by inducing a conformational change of the protein (Scicchitano et al., 2012). Different distinguishing properties of the native *I*_f_ are exhibited by the HCN isoforms, including activation by hyperpolarized membrane potentials, modulation by cAMP, permeability to Na^+^ and K^+^, and the chunk on the part of cesium (Cs^+^) (Ludwig et al., 2004).

## The *I*_f_ Channel As An Emerging Therapeutic Target

The funny channel is a good target for the development of new drugs as it generates spontaneous activity and controls the heart rate by acting specifically on the cardiac rate control and pacemaker rhythm. Lowering the heart rate under different cardiac conditions is one means of controlling CVDs pharmacologically, even though there are different risk factors (Sulfi and Timmis, 2006).

Various experimental and epidemiological studies have shown that, in spite of the multiple identified risk factors such as hypertension, diabetes, and smoking, an increased resting heart rate is one of the key independent predictors of CAD, heart failure, and cardiovascular mortality (Umana et al., 2003; Fox et al., 2008; Busseuil et al., 2010). An increased resting heart rate has also been linked to mental stress, which, in turn, aggravates myocardial ischemia in patients with CAD and is considered to be one risk factor for hypertension and atherosclerosis (Carter et al., 2005). The consequences of increased heart rate can favor the progression of myocardial ischemia as a result of mounting myocardial oxygen use and decreased coronary blood flow due to reduced diastolic filling time (Parker and Parker, 1998).

In mouse models of hypercholesterolemia and endothelial dysfunction, the administration of ivabradine reduced the levels of vascular oxidative stress markers and atherosclerotic plaque development and restored endothelial function through the inhibition of the *I*_f_ channel (Custodis et al., 2008). An increased heart rate can cause plaque rupture and associated myocardial problems (Dominguez-Rodriguez et al., 2011).

Heart rate during acute ischemia-reperfusion is an important determinant of susceptibility to reperfusion arrhythmias, with a higher heart rate found to cause a predisposition to arrhythmias in rats (Bernier et al., 1989). Bernier et al. (1989) found that pacing either throughout the experiment or during ischemia alone led to a rate-dependent increase in the occurrence of reperfusion-induced ventricular fibrillation (VF), from 25% in the unpaced hearts to >90% when the rate was ≥420 beats/min, but pacing during reperfusion alone did not increase the occurrence of reperfusion-induced VF. These data suggest that a clinical therapeutic strategy for heart rate reduction during ischemia-reperfusion may reduce the incidence of reperfusion arrhythmias. However, it remains unclear whether an increased heart rate is associated with ventricular or reperfusion arrhythmia. To investigate this, animal experiments have been performed using ivabradine to evaluate the outcome of selective heart rate reduction during the occurrence of ischemia and reperfusion reduced reperfusion VF. The heart rate reduction as a result of acute ischemia led to a reduction in the occurrence of reperfusion arrhythmias, whereas the heart rate at reperfusion alone did not influence the occurrence of reperfusion VF, as neither a bolus of ivabradine nor pacing prior to reperfusion changed the occurrence of reperfusion VF. Hence, the anti-arrhythmic effects of ivabradine in the heart rate reduction during acute ischemia may be the result of the slower development of ischemia-induced electrophysiological alterations (Ng et al., 2013).

**FIGURE 3:**
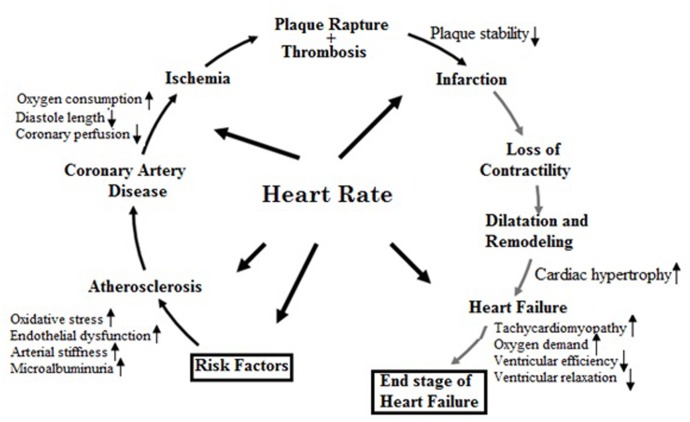
Pathophysiological effects of heart rate on the cardiovascular disease continuum (Adapted from Custodis et al., 2013 with permission).

All of the phases of the cardiovascular continuum (**Figure 3**) from vascular risk factors to cardiovascular episodes and heart failure are essentially influenced by heart rate. Hence, in CVD prevention, heart rate is one of the risk indicators and therapeutic targets (Kannel et al., 1987; Gillman et al., 1993; Diaz et al., 2005; Custodis et al., 2008, 2013; Fox et al., 2008).

Patients with chronic stable angina can benefit from decreased heart rate because of the associated improved myocardial perfusion and reduced myocardial oxygen demand. The use-dependence of heart rate reducing agents is one of their distinctive attributes in which the outcome of drug application accumulates through repetitive action (Van Bogaert et al., 1990). This important drug characteristic was achieved for ivabradine as a result of the accumulated inhibition of *I*_f_ current by the activation/deactivation procedure, which is therapeutically useful and it indicates that the slowing action of the drugs will be stronger at elevated heart rates when the effect of slower heart rate is essential (Bucchi et al., 2002).

According to a study conducted on mice to evaluate the effects of chronic mental stress and heart rate on endothelial function and cerebral ischemia, chronic mental stress impairs the function of endothelial cells, increases vascular and brain oxidative stress, and enlarges the size of cerebral lesions (Custodis et al., 2011). The endothelial function, oxidative stress, and ischemic brain injury could be restored, reduced, and protected, respectively, by heart rate reduction using ivabradine (10 mg/kg per day), as a result of the reduced activity of NADPH oxidase in the aorta in addition to aortic lipid peroxidation and reduction of the expression of nitric oxide synthase (eNOS). These results suggest that heart rate reduction by *I*_f_ inhibition is a likely new target for improving the cerebrovascular function after ischemic events (Custodis et al., 2011). In a separate study, Walcher et al. (2010) reported that the chemokine-induced migration of CD4-positive lymphocytes was inhibited upon administration of ivabradine by limiting both phosphatidylinositol 3 kinase (PI3K) activity and the phosphorylation of protein kinase B (AKT).

### *I*_f_ Channel Blockers

The previously established drugs for the treatment of angina have undesirable side effects in addition to reducing the heart rate, including the deterioration of peripheral vascular disease, psychological depression, bronchospasm, peripheral vasoconstriction of the extremities, hypotension, leg tiredness, and erectile dysfunction in the case of β-adrenergic blockers and peripheral edema, hypotension, and headache in the case of calcium-channel blockers (Sulfi and Timmis, 2006). Furthermore, the treatment of angina using the conventional therapies is often ineffective, with almost two-thirds of patients experiencing about two angina attacks per week on average (Pepine et al., 1994). Hence, a selective drug that reduces heart rate, such as ivabradine, will be necessary for some patients because of safety and efficacy issues (Izzo et al., 2012).

Numerous molecules that block the pacemaker channels have been developed (**Figure 1**), including alinidine, cilobradine, zatebradine, ZD7288, and ivabradine, but ivabradine (a benzocyclobutene-containing compound) is the only drug that has completed the full range of clinical trials and is currently marketed as an anti-anginal drug (under the brand name of Corlanor). The development of the other molecules was discontinued for various reasons, for instance, limited channel specificity for alinidine and ZD7288, a lack of substantial inotropic or vascular alterations for cilobradine, and the development of undesirable visual side effects for zatebradine (Bucchi et al., 2007).

The recently developed ivabradine acts specifically on the SAN as a novel selective heart-rate-reducing agent that selectively inhibits *I*_f_ channel binding, a primary SAN pacemaker current, and reduces the heart rate both at rest and during exercise (Bucchi et al., 2002; Borer et al., 2003). Ivabradine is currently licensed by the European Medicines Agency and the US Food and Drug Administration for the management of heart failure and stable angina both alone and in combination with other classes of drugs (Sulfi and Timmis, 2006; Tse and Mazzola, 2015).

### Therapeutic Effects of Ivabradine

The use of ivabradine to selectively reduce heart rate has been investigated in randomized trials such as the BEAUTIFUL and SHIFT trials, and heart rate was found to be an adjustable risk factor in patients with heart failure (Fox et al., 2008; Swedberg et al., 2010). In the BEAUTIFUL study, elevated heart rate (≥70 bpm) in patients with CAD and left ventricular dysfunction was a strongly independent risk factor, which was supported by the SHIFT study performed in a cohort of patients with a left ventricular ejection fraction of ≤35% and a heart rate of ≥70 bpm. Ivabradine did not improve the primary composite endpoint in either of these trials, but it was found to be significantly effective in a subgroup analysis of patients with a heart rate of ≥70 bpm and a left ventricular ejection fraction of ≤35%. Accordingly, this drug has been suggested as an add-on therapy in combination with beta blockers for patients with a heart rate of ≥70 bpm and an ejection fraction of ≤35%.

Additional trials have been performed in the search for safe and effective CAD treatments. A 3-month double-blind, multicenter, controlled trial conducted in patients with stable angina indicated that ivabradine treatment led to improved exercise tolerance without pharmacological tolerance or rebound phenomena, dose-dependent decreases in resting and exercising heart rate, a delayed onset of exercise-induced ischemia, and a decreased incidence of ambient angina attacks (Borer et al., 2003).

Milliez et al. (2009) performed a study to observe the beneficial effects of delayed ivabradine treatment in severe chronic heart failure in adult male Wistar rats. The results showed that ivabradine led to decreased mRNA and protein levels of cardiac angiotensin-converting enzyme, a marker of the cardiac renin-angiotensin-aldosterone system (RAAS), and angiotensin receptor 1, which indicated that the fibrotic remodeling of the remote myocardium was reduced due to the low level of RAAS activation. Thus, ivabradine allowed cardiac function to be retained after heart failure as a result of its heart-rate-reducing effects (Milliez et al., 2009). Another study that investigated induced dyslipidemia in the hypercholesterolemic rabbit model revealed that the levels of circulating angiotensin-II and aldosterone were correlated to the heart rate and significantly decreased upon ivabradine treatment (Busseuil et al., 2010).

The effects of ivabradine have also been compared with well-established beta blockers and calcium-channel blockers such as atenolol (Tardif et al., 2005), metoprolol (Becher et al., 2012), and amlodipine (Ruzyllo et al., 2007) in patients with stable angina or heart failure. These studies revealed ivabradine to have a comparable effectiveness to atenolol, with less myocardial depression, and amlodipine for the treatment of stable angina, although ivabradine increased the total exercise duration over the treatment period and was also associated with an increased time to limiting angina, time to onset of angina, and time to 1 mm ST-segment depression in comparison with amlodipine. Although metoprolol reduced the heart rate, it did not prevent the decline in cardiac function and undesirable remodeling, in spite of a reduction in the inflammatory stress response, whereas ivabradine exhibited additional useful cardiac effects that contribute to preventing heart failure.

The mechanism of the reduction in heart rate mediated by ivabradine through the selective inhibition of the funny channel is different to those of beta blockers and calcium-channel blockers, the two conventionally prescribed anti-anginal drugs. One of the distinct characteristics of ivabradine is that it is not intrinsically voltage-dependent but instead depends on the ion flow pathway across the channel pore, i.e., its inhibition of the funny channel is current dependent (Bucchi et al., 2002). *In vitro* animal models as well as clinical trials have shown that ivabradine selectively inhibits the *I*_f_ channel at very low concentrations that cannot possibly affect the L- and T-type Ca^2+^ channels or delayed outward K^+^ channels (Sulfi and Timmis, 2006).

According to the study conducted by Chen et al. (2014) in diabetic mice, ivabradine attenuates apoptosis, slows down the expression and action of matrix metalloproteinase 2, and hence improves the cardiac function of the animals. This finding indicates that ivabradine may also benefit diabetic patients.

Cardiac fibrosis formation and progression, where TNF-α plays an important role, is implicated in inflammation, which plays a serious role in heart failure (Fang et al., 2017). A number of patients with heart failure may develop cardiac fibrosis and it is well known that myocardial infarction induces cardiac remodeling, including fibrosis of the remote myocardium and myocyte hypertrophy (Pfeffer and Braunwald, 1990). Based on this concept, Fedorov (2015) assessed the antifibrotic activity of ivabradine in rat myocardium in the case of chronic heart failure and the results revealed that ivabradine decreases ventricular interstitial fibrosis. According to Becher et al. (2012), ivabradine could decrease cardiac fibrosis through the inhibition of inflammatory responses and cardiac apoptosis.

Another study indicated that ivabradine may be a promising agent for the management of patients with viral myocarditis (Yue-Chun et al., 2016). According to this report, ivabradine inhibited the p38 MAPK pathway, downregulated inflammatory reactions, and reduced collagen expression in mice infected with chronic viral myocarditis induced by coxsackievirus B3, thereby slowing down the progression of viral myocarditis to dilated cardiomyopathy.

Ivabradine is generally well tolerated (Tardif, 2007), but atrial fibrillation (Fox et al., 2014; Cammarano et al., 2016), excessive bradycardia (Demontis et al., 2009; Fox et al., 2014; Cammarano et al., 2016), phosphene (Thollon and Vilaine, 2010; Fox et al., 2014; Cammarano et al., 2016), drug-related nuisances, and blurry vision (Cammarano et al., 2016) were the prominent side effects reported by the clinical trial patients who withdrew from the studies. Ivabradine is also metabolized by CYP3A4 enzymes and it may possibly interact with enzyme inhibitors such as ketoconazole, erythromycin, diltiazem, or verapamil (Yu et al., 2016).

### Current Controversies Concerning Ivabradine

Multiple randomized controlled trials have been conducted for ivabradine, such as the BEAUTIFUL, SHIFT, SIGNIFY, and ASSOCIATE studies, which involved different disease conditions (mainly heart failure and stable angina). However, these studies revealed several inconsistencies. Although most of the randomized trials found a positive effect for ivabradine, mainly in terms of treating stable angina for patients with heart rates of >70 bpm (Borer et al., 2003; Ruzyllo et al., 2007; Fox et al., 2008; Tardif et al., 2009), the current evidence indicates that the inclusion of ivabradine in the regular background therapy of patients with stable CAD devoid of clinical heart failure did not improve patient outcomes (Fox et al., 2014), whereas another study reported the non-inferiority of ivabradine compared with the standard care including beta blockers (Tardif et al., 2005). This indicates that the evidence remains controversial.

A recent meta-analysis based on randomized trials of stable CAD patients indicated that the non-selective utilization of ivabradine is not supported by attestation and can be linked with adverse effects such as new-onset atrial fibrillation, bradycardia, and drug-related irritation (Cammarano et al., 2016). Therefore, the use of ivabradine for CAD seems to be questionable in terms of the cohort of patients treated and this deserves more rigorous investigation in the future.

A study by Walcher et al. (2010) indicated that ivabradine also inhibits other sites, which suggests another controversy regarding its site of action. This report revealed that ivabradine inhibits the chemokine-induced migration of CD4-positive lymphocytes by limiting both PI3K activity and the phosphorylation of Akt. PI3K/Akt is an important molecule that was found to be down-regulated in type 2 diabetes mellitus in insulin signaling (Jiang and Zhang, 2002). However, ivabradine has shown inconsistent results in terms of the instruction of the PI3K/Akt transduction lane, in particular, up-regulating eNOS expression independently of the PI3K/Akt pathway (Borer, 2004). Therefore, the different inhibitory effects of ivabradine require further consideration.

### Clinical Relevance of the *I*_f_ Channel

The main function of the HCN channels is generating the sinus rhythm and hence controlling the heart rate. Dysfunction in the funny channels would be expected to cause arrhythmic behavior. Mutations of the HCN channel genes and their effects on cardiac function have been investigated by molecular approaches (Scicchitano et al., 2012).

Mutations in HCN-2 and HCN-4 were found to slow down the pacemaking activity of the SAN (Baruscotti and Difrancesco, 2004). To date, mutations of the human *I*_f_ channel has been restricted to HCN-4 (Schulze-Bahr et al., 2003; Laish-Farkash et al., 2010; Duhme et al., 2013) or the potassium voltage-gated channel subfamily E member 2 (KCNE2), also known as minK-related peptide 1 (MiRP1), is a protein that is encoded by the *KCNE2* gene on chromosome 21 (Nawathe et al., 2013). Voltage clamp experiments, a technique to measure the ion currents passing through the membranes of excitable cells such as neurons while holding the membrane voltage at a fixed level, were performed on wild-type and mutant human HCN-4 channels expressed in Chinese hamster ovary (COS-7) cells, *Xenopus* oocytes, or human embryonic kidney (HEK-293) cells and revealed modifications in the appearance or kinetics of mutant HCN-4 channels. However, the extents to which these transformations affect the *I*_f_ channel flowing in a human SAN action potential remains unclear. The HCN-4 mutation caused both inherited sinus bradycardia, death during early embryogenesis, and other rhythmic disturbances in experimental animals, which clearly indicates the relevance of the funny channels in the disturbance of the cardiac rhythm by gene- or cell-based therapeutic approaches (Barbuti and DiFrancesco, 2008).

The experimental evidence on the major outcomes of the loss-of-function mutations in HCN-4 and KCNE2 reveals inconsistencies between the *in vitro* and *in silico* data and the clinically observed data in the *I*_f_ channel in terms of the action potential and current flow under both exercise and resting conditions, which points to possibilities for future research (Verkerk and Wilders, 2014). In general, HCN-4 and to a lesser extent HCN-2 are the predominant components of the *I*_f_ channel and may lead to future applications in the treatment of rhythmic disturbances, replacing the current approach of using electronic devices with the use of cell/gene-based therapy to deliver the correct genes that encode the *I*_f_ channels.

## Conclusion

Multiple studies have demonstrated that atherosclerosis, heart failure, CAD, stroke, and arrhythmia are self-regulating prognosticators of CVDs as a result of an elevated heart rate. Even though randomized clinical trials have presented some controversies regarding the clinical use of ivabradine for CAD, the drug provides a safe and effective means to reduce heart rate, either alone or in combination with the already existing drugs, by selectively inhibiting the *I*_f_ channel without significantly affecting the action potential, inotropic activity, or ventricular contractility of the heart and with minimal adverse effects. Currently, funny channel inhibition is being targeted for the treatment and prevention of CVDs such as atherosclerosis and stroke.

## Future Perspectives

Based on the available evidence and the prospect of *I*_f_ channel targeting; it is very apparent that inhibition of this channel can be used for the prevention and treatment of cardiovascular-related mortality and morbidity. Through this strategy, it seems plausible to reduce CVD morbidity and mortality by an early screening of the at-risk population by measuring the simple vital sign of resting heart rate and providing a long-acting dosage form of the current oral or intravenous *I*_f_ inhibitors. Furthermore, the *I*_f_ channel has recently been investigated for the prevention and treatment of stroke, hypertension, atherosclerosis, myocardial infarction, and arrhythmia; fortunately, all of the *in vitro* studies and animal models showed positive findings. It is also suggested that the use of cell/gene-based therapy for the *in situ* delivery of the genes that encode the *I*_f_ channel might represent a future treatment strategy for rhythmic disturbances, which could replace the current practice of using electronic devices. Hence, in the future, *I*_f_ channel inhibitors might be clinically useful for prevention and treatment of these disorders.

## Author Contributions

HM and TT conceived the review and wrote the draft manuscript. TT, HM, and MB participated in improving the draft manuscript and writing the final manuscript. All of the authors approved the final manuscript.

## Conflict of Interest Statement

The authors declare that the research was conducted in the absence of any commercial or financial relationships that could be construed as a potential conflict of interest.
